# Characterisation and modelling of ductile fracture in incremental sheet forming of an aluminium alloy with consideration of temperature variations

**DOI:** 10.1007/s12289-025-01970-z

**Published:** 2025-12-26

**Authors:** Zhenyuan Qin, Shakir Gatea, Hengan Ou

**Affiliations:** Department of Mechanical, Materials and Manufacturing Engineering, Faculty of Engineering, The University of Nottingham, Nottingham, NG7 2RD UK

**Keywords:** Incremental sheet forming, Microscopic analysis, Ductile fracture behaviour, GTN damage model, Void evolution, Finite element method

## Abstract

Although extensive studies have been conducted to predict ductile fracture at room temperature, no attempt has been made to use Gurson–Tvergaard–Needleman (GTN) model to capture the effect of temperature due to spindle speed induced friction heat on damage accumulation in incremental sheet forming (ISF). In this work, the temperature effect is incorporated into a shear modified GTN model to characterise ductile fracture behaviour of an aluminium alloy (AA1050) at varying temperatures in ISF processing under different strain states. The correlation between void evolution and damage accumulation at different temperatures is revealed, and the void volume fraction (VVF) at different deformation stages is determined by conducting microscopic in-situ tensile test. By introducing a new temperature-dependent VVF function and determining appropriate GTN damage parameters, ductile fracture under different ISF processing temperatures is evaluated using FE simulation and validated by ISF experimental testing. The results show that the proposed temperature-dependent VVF function in GTN modelling is capable of predicting the ductile fracture of both the set temperatures in in-situ tensile tests and the varying temperature conditions in ISF processes. This study unveils the underlying mechanisms behind material deformation and damage evolution with consideration of temperature variations caused by tool spindle speed at various ISF forming conditions, which provides guidance for process optimisation and formability improvement in ISF process.

## Introduction

Incremental sheet forming (ISF), or single point incremental forming (SPIF) is recognised as a promising manufacturing technology and has drawn increasing attention in the past decades. In ISF, a hemispherical forming tool moves along a pre-defined trajectory to progressively impose cyclic and localised deformation on the blank sheet until the desired geometry of part is achieved [1]. In recent years, several variants of ISF have been developed including two-point incremental forming (TPIF) [2, 3] and double-sided incremental forming (DSIF) [4, 5] to further improve the process stability and forming accuracy. Both conventional ISF and its variants have considerable advantages such as high flexibility and adaptability, low tooling costs, high energy efficiency and improved material formability, and are accordingly considered as a viable manufacturing process for practical applications in automotive [6], aerospace [7] and biomedical field [8]. However, question still remains on the underlying mechanism of deformation and fracture and its intrinsic relationship with formability especially when the temperature effect plays an important role in ISF processing. Extensive studies have been conducted to reveal the deformation mechanism and fracture behaviour of ISF process. Silva et al. [9] proposed a theoretical model based on membrane analysis to investigate the fundamentals behind material fracture. They demonstrated that the fracture occurs as the result of meridional tensile stress instead of in-plane shear. Jackson and Allwood [10] found that multiple deformation modes including stretching, bending and shear have effects on the forming limit in ISF. By carrying out continuous bending under tension test, Hadoush et al. [11] pointed out that bending plays a significant role in stabilising the deformation under tension state, and an enhanced deformation stability can be achieved due to the existing of compressive stress. Malhotra et al. [12] employed FEA fracture modelling to predict the damage evolution in ISF of AA5052 sheet. The results indicated that the damage accumulation depended on both hydrostatic stress and through-thickness shear in the plane parallel to the tool motion direction. An analytical evaluation by Fang et al. [13] suggested that the deformation and yielding occurred not only in the tool-workpiece contact area, but also in the adjacent inclined wall undergone plane strain stretching. Ai et al. [14] developed an analytical model with consideration of bending deformation and strain hardening, and concluded that these two factors had close correlation with deformation stability and material formability, in which bending was a major cause of the improved ISF formability while the effect of strain hardening was less obvious. By analysing the stress state in the localised deformation zone, Lu et al. [15] identified the role of through-the-thickness-shear during ISF process. It was suggested that the higher shear stress would facilitate the deformation stability but reduce formability at the same time. Through experimental observation, Franzen et al. [16] concluded that there are three typical failure modes including crack along circumferential direction (Mode 1), wrinkling along the inclined wall (Mode 2) and tearing along the radial direction (Mode 3) in ISF. Fernández et al. [17] investigated the deformation and failure mechanics in SPIF of AA2024-T3 sheets, and found that failure by wrinkling occurred when the minor principal compressive stress reached a critical level. Chang and Chen [18] applied membrane analysis with a generalised ductile fracture criterion to determine the influence level of several deformation mechanisms (bending, shearing and cyclic loading) on fracture strain under different ISF processing conditions. Wu et al. [19] indicated that the damage accumulation is essentially attributed to a resultant effect of sheet thickness thinning and strain concentration induced by circumferential contact angle.

Material formability and fracture behaviour can be characterised by using damage models. Basak et al. [20] implemented the Hill48 anisotropy plasticity theory into seven ductile fracture models and demonstrated that the BW fracture model provided the most accurate prediction of forming limit and captured the effect of anisotropy on the fracture strain of AA6061 sheet metal. Zhan et al. [21] developed a coupled numerical–analytical framework integrating temperature and strain rate dependent ductile fracture criteria to predict the failure behaviour of AA2024-T3, identifying the role of high-speed tool rotation in the enhanced formability in ISF. Chang and Chen [18] established an analytical stress triaxiality model combined with Hu’s ductile fracture criterion to predict the fracture strain and determine the dominant deformation mechanism in ISF. Wierzbicki and co-workers developed a unified ductile fracture locus that incorporated both stress triaxiality and Lode angle to describe the equivalent strain to fracture across a wide range of loading paths, providing a continuous transition among tension, shear and mixed failure modes with high predictive capability and relatively low calibration effort [22–25]. In the framework of ductile damage modelling, a micromechanical damage criterion, which treats the damage indicator as the ratio of void volume and representative element volume, is widely used to predict the ductile fracture behaviour of porous materials. Gurson [26] firstly developed a micromechanics-based constitutive model to incorporate the effect of void growth and hydrostatic stresses on the ductile fracture. Later, Tvergaard [27] and Tvergaard and Needleman [28] extended the original Gurson model to better describe the voids interaction and its effect on the void coalescence and subsequent material crack. This modified model, referred to as Gurson–Tvergaard–Needleman (GTN) damage model, attributes the ductile fracture phenomenon to the mechanism of nucleation, growth and coalescence of microvoids. Extensive research has been published on the application of GTN model to metal forming processes. Kami et al. [29] utilised anisotropic GTN model coupled with Hill’48 quadratic yield criterion to numerically determine the forming limit curves of AA6016-T4 in Nakajima test. The results showed that limit strains predicted by the GTN model are better consistent with experimental results especially under the biaxial tension state than that evaluated using Marciniak–Kuczynski and modified maximum force criterion models. Teng et al. [30] investigated the ductile fracture behaviour in Hydro-bulging of aluminium alloy semi-ellipsoidal shell based on GTN model. It was noted that model prediction results regarding bursting position, bursting pressure, and the maximum dome height were in good agreement with experimental data. Ying et al. [31] implemented shear-modified GTN model into Abaqus/explicit to predict occurrence of rupture in quasi-static small punch test of high strength steel 22MnB5. The authors demonstrated that the model was able to predict sheet metal formability under wide range of stress triaxiality. In the research of Nasir et al. [32], by coupling bifurcation-based criteria with GTN damage model, different material plastic instabilities including either diffuse or localised necking and ductility limits of sheet metals under various strain loading paths can be well predicted. Yildiz and Yilmaz [33] used GTN model to numerically explore the influence of heat treatments on the formability of AA6061 sheets with different thickness in deep drawing process. They suggested that the sheet thickness had a positive effect on the forming limit, and the fracture strain would exhibit a drop as duration of heat treatment increased.

To characterise the fracture behaviour in ISF, Li et al. [34] employed GTN model integrating with Hill’48 anisotropic yield criterion to predict damage evolution and ductile fracture of DC06 sheet at the microscopic level. It was noted that void volume fraction could be an effective index to evaluate material formability. Chang and Chen [35] modified the original GTN model by considering the effect of oriented void cluster on the void coalescence. They found that voids tended to coalesce and grow along meridional direction instead of thickness direction, and void coalescence mechanism can be well reflected by GTN model. Gatea et al. [36] deployed a shear modified GTN model to predict the ductile fracture behaviour of pure titanium in single point incremental sheet forming. The prediction results indicated that the shear effect played a significant role under meridional tensile stress to promote fracture occurrence, and the maximum forming depth obtained from model showed good agreement with experimental results. In their subsequent work [37], based on experimental and numerical investigations, they concluded that the effect of forming parameters, namely step size, feed rate and tool size, on the forming limits, strain evolution as well as thickness distribution can also be represented by shear modified GTN model. Kumar et al. [38] adopted GTN model to determine the role of residual hydrostatic stress on material damage in ISF. It was highlighted that an increase in residual hydrostatic stress would accelerate localised necking and crack initiation. A modified GTN model taking into account the Lode angle effect proposed by Peng and Ou [39], was able to achieve improved prediction accuracy of damage evolution under changing deformation modes in single point and double-sided incremental forming. This was because the new model provided more precise evaluation of the material softening and damage accumulation due to shear and it was compensated for by the coverage of shear and tension-under-compression cases.

Although above literature has proven the applicability of GTN damage modelling to the prediction of material failure in ISF, there is a notable lack of research in predicting ductile fracture with the consideration of the effect of temperature due to spindle speed induced friction heat in ISF processing. It is well known that the forming limit of material is directly correlated with temperature variation when the spindle speed of the forming tool is applied in ISF. Xu et al. [40] explored the effect of spindle speeds on the maximum drawing angle of AA5052-H32 alloy in ISF. The results showed that the maximum drawing angle at spindle speed of 7,000 rpm was raised by 20.6% compared to the case without spindle speed. Similarly, Wang et al. [41] found the spindle speed provided a positive influence on the formability of AA2024 and AA5052 alloys. Khalatbari and Lazoglu [42] experimentally demonstrated that the poor formability of Polyoxymethylene (POM) at room temperature can be improved by using a specific level of spindle speed to produce suitable forming temperature. Wu et al. [43] applied ISF to synchronous bonding of steel and aluminium alloy sheets. According to their findings, the spindle speed induced friction heat can not only reach the optimal forming depth, but also greatly improve the interfacial bonding strength of laminated parts. Grün et al. [44] utilized one-factor-at-a-time (OFAT) and design-of-experiment (DoE) methods to study the forming limit of Ti6Al4V alloy in ISF. They stated that the spindle speed played a dominant role in temperature increase and thus enhanced formability, which was also confirmed in [45] by performing the experiment on the same material. Zhu and Ou [46] developed a new heat-assisted friction stir incremental sheet forming process to form hard-to-deform thermoplastics, highlighting that the use of spindle speed is conducive to reducing forming force and maintain uniform through-thickness temperature distribution and thus achieve the targeted forming depth. Long et al. [47] found that the spindle speed is an important factor affecting frictional heating and vibration frequency, and spindle speed induced thermal and vibration softening is the key mechanism leading to improved formability during rotational vibration-assisted incremental sheet forming. Although above experimental observations reflect the role of forming temperature in material formability in ISF, how to accurately predict the forming limit at varying temperatures induced by spindle speeds in ISF is still an outstanding question. Therefore, it is necessary to develop an approach of damage modelling with the consideration of the effect of temperature.

The review of the aforementioned literature indicates that the deformation and fracture mechanisms considering the temperature effect under different forming conditions during ISF process are still yet to be revealed. How to capture the role of spindle speed in temperature increase and corresponding formability enhancement, and to reflect the correlation between void evolution and damage accumulation at varying temperatures in ISF are unclear. To fulfil the above research gaps, this work aims to incorporate the temperature effect via a new temperature-dependent VVF function into shear modified GTN model for the first time so that the fracture evolution can be accurately evaluated at varying forming temperatures in ISF processing under different strain states. The model was implemented into finite element Abaqus/explicit to capture the influence of temperature on the fracture initiation in ISF under different spindle speeds. The fracture depth and position obtained from FE simulations were validated by ISF experimental results. The findings from this study provide an in-depth understanding of material deformation and a new approach to predict damage mechanisms with consideration of temperature effect caused by tool spindle speed and under different ISF processing conditions.

## Gurson-Tvergaard-Needleman (GTN) model

From the micromechanical perspective, the ductile fracture of metallic materials is controlled by the mechanism of void nucleation, growth and coalescence. The void evolution during deformation and its correlation with damage accumulation can be described by GTN model. The yield surface of the GTN model is defined as:

1 $$\varnothing=\left(\sigma_q/\sigma_y\right)^2+2q_1f^\ast\cos h\left(-3q_2p/\left(2\sigma_y\right)\right)-\left(1+q_3f^{\ast2}\right)=0$$

where $\:{\sigma\:}_{q}$ is the equivalent Von Mises stress, $\:{\sigma\:}_{y}$ is the flow stress of the fully dense matrix material, $\:{q}_{1}$, $\:{q}_{2}$ and $\:{q}_{3}$ are the constitutive parameters that accounts for void interaction effects, $\:p$ is the hydrostatic stress. $\:{f}^{*}$ is used to describe the final material failure for void coalescence, which is given by:

2 $$f^\ast=\left\{\begin{array}{cl} f&if\;f\;\leq\;f_c\\f_c+\left(\left(\overline{f}_F-f_c\right)/\left(f_f-f_c\right)\right)\left(f-f_c\right)&if\;f_c<f<f\\\overline{f}_{F}&{{if\;f\geq f_f}}\end{array}\right.$$

where $\:f$ is the void volume fraction defined as a ratio of the volume of voids to the total volume of the matrix material. $\:{f}_{c}$ is the void volume fraction at critical stage, $\:{f}_{f}$ is the void volume fraction when the material has completely lost its stress-bearing capacity. Since the temperature exerts a significant effect on material formability during ISF, the void volume fractions at different deformation stages can be defined as a function of temperature to capture the fracture behaviour of material at different forming temperatures. $\:{f}_{i}$ can be expressed in a general form:

3 $$\:\begin{array}{c}f_i=f\left(\epsilon\:,\:T\right)\end{array}$$

where $\:{f}_{i}$ is the void volume fraction at the different stages of deformation, e.g., nucleation, critical or fracture stage; $\:\epsilon\:$ and $\:T$ are the strain and temperature.

When the shear loading is introduced, the void evolution at low or negative stress triaxiality should be considered. Therefore, Nahshon and Hutchinson [48] proposed an expression to predict the increase in the rate of void due to low or negative stress triaxiality. This mechanism showed a good evaluation of damage contribution of shear in ISF [35, 36, 49].

4 $$\:\begin{array}{c}{df}_{shear}=k_{\omega\:}(f\omega\:\left(\sigma\:\right)/{\sigma\:}_q)S:\dot{\epsilon\:}^p\end{array}$$

Parameter $\:{k}_{\omega\:}$ is used to quantify the shearing effect during deformation. After incorporating the shear mechanism into the original GTN model, the total void volume fraction can be expressed as follows:

5 $$\:\begin{array}{c}f_{total}={f_{initial}+(df}_{nucleation}+{df}_{growth}+{df}_{shear})\end{array}$$

## Experimental testing

### In-situ tensile test and characterisation of damage evolution

**Fig. 1:**
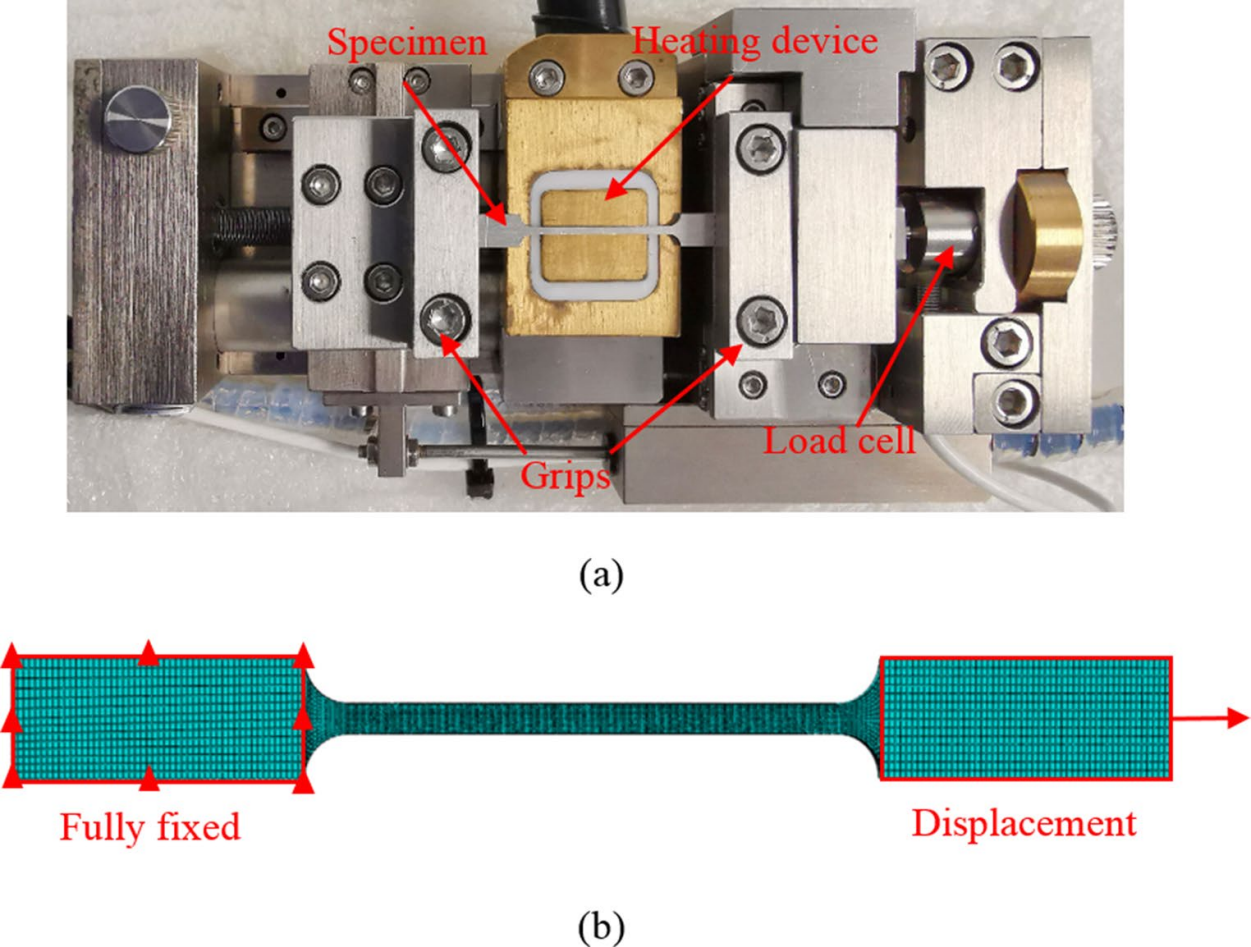
Experimental setup and FE model for in-situ tensile test. (**a**) tensile test equipment showing the clamping, heating and force measurement sections. (**b**) FE model of the tensile test showing the boundary conditions of simulation

To reveal the void evolution during deformation at different temperatures, in-situ tensile test was performed on aluminium alloy AA1050 smooth tensile specimens at three temperature conditions (25 °C, 85 °C and 145 °C). AA1050 aluminium alloy was selected in this study due to two reasons. First, AA1050 has been widely used in industrial applications due to advantages such as high corrosion resistance, thermal conductivity and ductility. Second, AA1050 is commonly employed in ISF focused research because of good formability, which is benefited to obtain formed parts with target shapes and forming depths and thus better investigate the deformation and fracture behaviour under various ISF forming conditions. The test equipment used is a JEOL JSM-7100 F microscope with a micro tensile testing module, which can be used to not only determine the flow behaviour of material, but also measure the microstructural morphology of the sample in real time. The dimensions of tensile specimens were designed to a total length of 40 mm, gauge length of 17 mm, gauge width of 1 mm and thickness of 0.7 mm. In testing, the specimen was first fixed into the grips and its tensile direction was kept in parallel to the loading direction of equipment, as shown in Fig. 1(a). The machine chamber was pumped down to a vacuum pressure of approximately 2.3 × 10^− 3^ Pa to produce a consistent electron beam. The sample was heated up to a specified temperature at a heating rate of 1 °C/s. Once the target temperature was reached, the undeformed condition of sample was held for 5 min to ensure uniform temperature distribution. Then, the sample was stretched with a traverse velocity of 0.5 mm/min at the target temperature until it broke and the corresponding stress-strain curve was generated. Each test was repeated three times to achieve the required repeatability of the experimental results.

In this work, the void volume fraction was determined following the methodology outlined in both BRITISH STANDARD BS 7590 [50] and ASTM E562-02 Standard [51]. This involves measuring the voids area at different stages of deformation to calculate the void volume fraction. This method is considered as an effective means to quantify void volume fraction (VVF). The accuracy of such method is due to the GTN damage model’s fundamental assumption that voids maintain a spherical shape during deformation process, aligning well with this approach. Since the entire deformation process of in-situ tensile tests was observed by scanning electron microscope (SEM), the damage parameters, i.e., the void volume fraction at different deformation stages ($\:{f}_{0},\:{\:f}_{n},\:{\:f}_{c},\:\:{f}_{f})$ of GTN model can be determined during the test. The initial void volume fraction ($\:{f}_{0}$) can be identified by scanning the specimen surface to quantify the proportion of initial voids prior to deformation. When plastic straining happens, voids tend to nucleate from the inclusions or second phase particles by debonding or cracking, so the nucleation void volume fraction ($\:{f}_{n}$) can be evaluated at this stage. The critical void volume fraction ($\:{f}_{\mathrm{c}}$) corresponds to the moment when the tensile stress reaches the maximum value and the necking phenomenon occurs. After the occurrence of necking the material progressively loses its load bearing capacity until fracture. The fracture void volume fraction ($\:{f}_{f}$) can be examined by conducting the fractographic analysis of samples to determine the percentage of voids in the fracture surface.

### Incremental sheet forming (ISF) testing

**Fig. 2:**
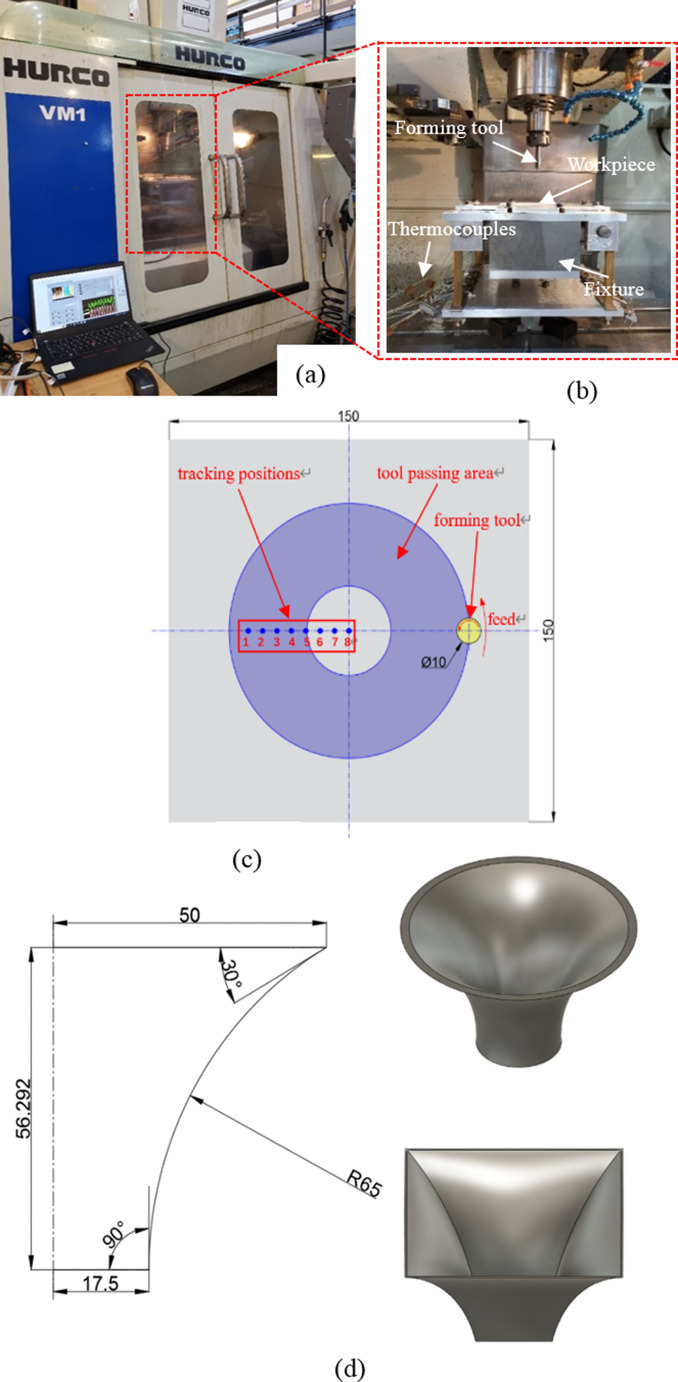
Experimental setup, CAD models and geometrical details of incremental sheet forming (ISF) parts. The figures shows, (**a**), (**b**) the experimental setup, (**c**) tracking positions of thermocouples, (**d**) forming profiles of the hyperbolic truncated cone and truncated pyramid parts with varying drawing angles from 30° to 90° (unit: mm)

The ISF testing was carried out on a HURCO-VM1 three axis CNC milling machine shown in Fig. 2(a) and (b) where an adjustable fixture was assembled to fasten the blank sheet. The workpiece used is AA1050 aluminium alloy with the size of 150 × 150 × 0.7 mm. Table 1 shows the mechanical and thermal properties of the workpiece. In order to investigate the fracture behaviour and formability of material under various forming conditions, hyperbolic truncated cone and hyperbolic truncated pyramid shapes with varying drawing angles ranging from 30° to 90° were designed to be made by employing a high-speed steel hemispherical ball nose tool with a radius of 5 mm moving along a pre-defined tool path which was conformal to the target part geometry. The desired forming depth is 56.3 mm to attain a maximum drawing angle of 90° for maximum formability. Figure 2(d) presents the geometrical details of the two designed shapes. The experiments were conducted with two levels of spindle speeds (0 rpm and 2,500 rpm), feed rate of 1,500 mm/min and step down of 0.5 mm. Eight K-type thermocouples were fixed on the underside of the workpiece with respect to the side contacted with the forming tool to record the forming temperatures at different tracking positions with equal spacing of 6 mm, as shown in Fig. 2(c). Rando HD68 oil was applied to the contact surface as a lubricant to reduce the wear between the forming tool and workpiece. The tests were stopped when the fracture occurred or until the finish of tool path program. The vertical distance between the flange and fracture location was defined as the fracture depth for formability evaluation.

**Table 1:** The material properties of AA1050 at room temperature [52, 53]

Material	Density [kg/m^3^]	Poisson’s ratio	Young’s modulus [GPa]	Yield stress [MPa]	Specific heat [J/(kg.K)]	Conductivity [W/(m.K)]
AA1050	2,700	0.33	71	90	900	231

## Finite element (FE) modelling

### Tensile test simulation

In this work, the uniaxial tensile simulation was conducted by using Abaqus/explicit to validate the accuracy of experimentally obtained GTN damage parameters. The schematic of FE model of uniaxial tensile test is shown in Fig. 1(b). Based on experimental setup, the left side of the sample is fully fixed whilst a displacement boundary condition is applied to right side of the sample. Due to the deformation propagation and damage accumulation, a higher density of mesh with element size of 0.1 mm was applied to the central gauge region. In the clamping region, coarse mesh with element size of 0.2 mm is defined to reduce computation cost. Both regions were discretised with the solid elements, 8-node linear brick, reduced integration and hourglass control (C3D8R) with using central-difference explicit time integration scheme. The total number of elements is 16,464. The material properties shown in Table 1 are used as input date of FE model. The determined GTN damage parameters under each temperature condition are implemented into FE simulation to generate the true stress strain curves which are used for comparison with experimental flow curves.

### Incremental sheet forming (ISF) simulation

Fully coupled thermo-mechanical simulation of ISF process of AA1050 was performed using Abaqus/explicit solver. The forming tool was considered as an analytical rigid body while the blank sheet was modelled as a deformable part. All degrees of freedom are fully constrained to the clamping region of the workpiece whilst its central forming area was free to deform as per the experimental condition. In order to resemble the boundary conditions of forming tool in tests, the tool movements were controlled by the displacement amplitudes with respect to time along X, Y and Z directions, and the tool rotation was governed by an angular velocity relative to the reference point of the tool. The experimental stress-strain data at different temperatures presented in Fig. 9 was implemented into FE to describe the hardening behaviour of workpiece. The isotropic von Mises yield criterion was used in FE modelling. To achieve the balance between simulation accuracy and computation efficiency, the stable time increment was defined by a mass scaling of $\:2\times\:{10}^{-5}\:s$. The fracture behaviour and failure position under various forming conditions were predicted by GTN damage model which was implemented into Abaqus/explicit package via a user-defined material subroutine VUMAT. To simulate the damage accumulation during the process, an element deletion approach was employed to check where the forming limit was reached so that the elements were deleted with all integration points losing the stress carrying capacity. Enhanced hourglass control was used to improve the element stability under the bending load. For the sake of simulation accuracy with acceptable computing time, a refined mesh with an element size of 0.5 mm and a coarse mesh with a maximum element size of 5 mm were created inside and outside of the central forming region of the sheet, respectively. The mesh size was chosen based on a detailed analysis of mesh size effect from previous work [36, 49] that employed the GTN model for ductile fracture prediction in ISF at room temperature. Three layers of elements were defined through the thickness of the blank sheet. Both regions were discretised with eight-node thermally coupled solid brick element with reduced integration and hourglass control (C3D8RT) with using central-difference explicit time integration scheme. The total number of elements is 118,385. The material parameters tabulated in Table 1 were used as input data. Tool-workpiece contact interaction was defined by surface-to-surface friction algorithm in conjunction with penalty contact formulation. The coefficient of friction between the forming tool and the sheet was set to 0.09 by referring to a previous work [54]. According to thermo-mechanical theory, the total heat generation was ascribed to the combined effects of interfacial friction and inelastic deformation, in this work, it was assumed that 100% of frictional dissipation and 90% of plastic work were converted into heat [55–57]. In terms of thermal boundary conditions, the initial temperature of the workpiece was set to 20 °C in accordance with the tests. Additionally, the central forming region was applied with a convective heat transfer coefficient of 20 W/(m^2^.K) [58] to describe the heat loss due to natural convection, while the flanging region was imposed with a thermal contact conductance coefficient of 1,000 W/(m^2^.K) [59] to define the heat conduction between the fixture and the sheet.

## Results and discussion

### Microscopic characterisation of void evolution of in-situ tensile samples

**Fig. 3:**
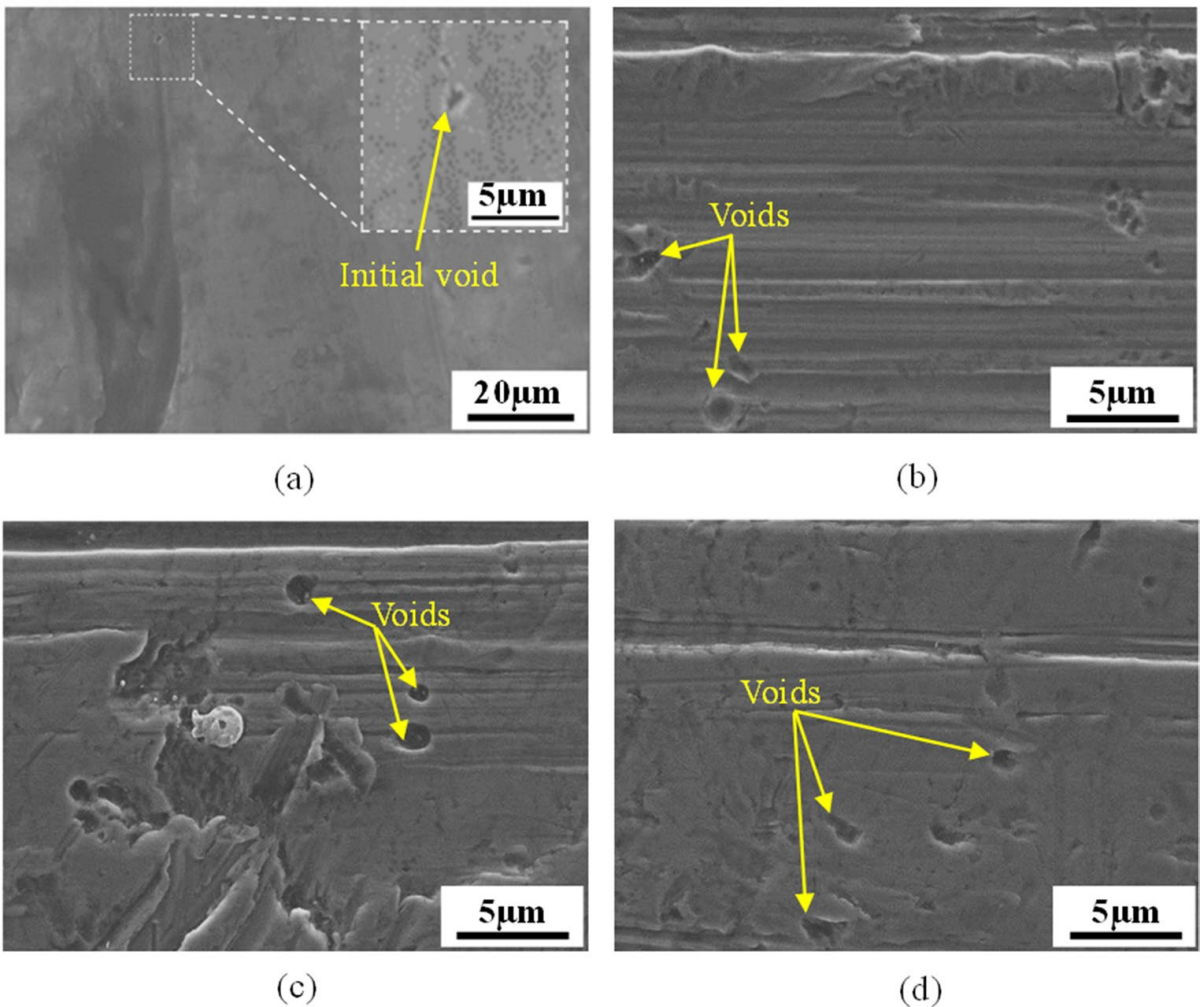
Microscopic morphology of tensile specimen at the initial and nucleation stages. (**a**) Microscopic morphology of tensile specimen at the initial stage before deformation. (**b**), (**c**) and (**d**) Microscopic morphology of tensile specimen at the nucleation stage at 25 °C, 85 °C and 145 °C, respectively. This shows a clear indication that the initial void with small size is inherent to the tensile specimen, and the newly nucleated voids retain their initial shape and size without further growth or interaction with surrounding voids

As the deformation propagates during the in-situ tensile test, voids inside the material sequentially undergo nucleation, growth followed by coalescence and subsequent micro-crack. This void evolution was experimentally characterised by using scanning electron microscope (SEM) combined with energy-dispersive x-ray spectrometry (EDS). Figure 3(a) depicts the microscopic image of the micro tensile specimen at the initial stage before deformation. It is observed that the initial void with small size is inherent to the material. When the displacement is increased leading to the occurrence of plastic yielding, new voids begin to nucleate from an inclusion or second phase particle via debonding or decohesion that happen when the peeling stress at particle/matrix interface exceeds the interfacial cohesive strength [60]. The nucleation stage at different temperatures is microscopically shown in Fig. 3(b)-(d). It can be seen that the newly nucleated voids keep their initial shape without further growth or interaction with surrounding voids.

**Fig. 4:**
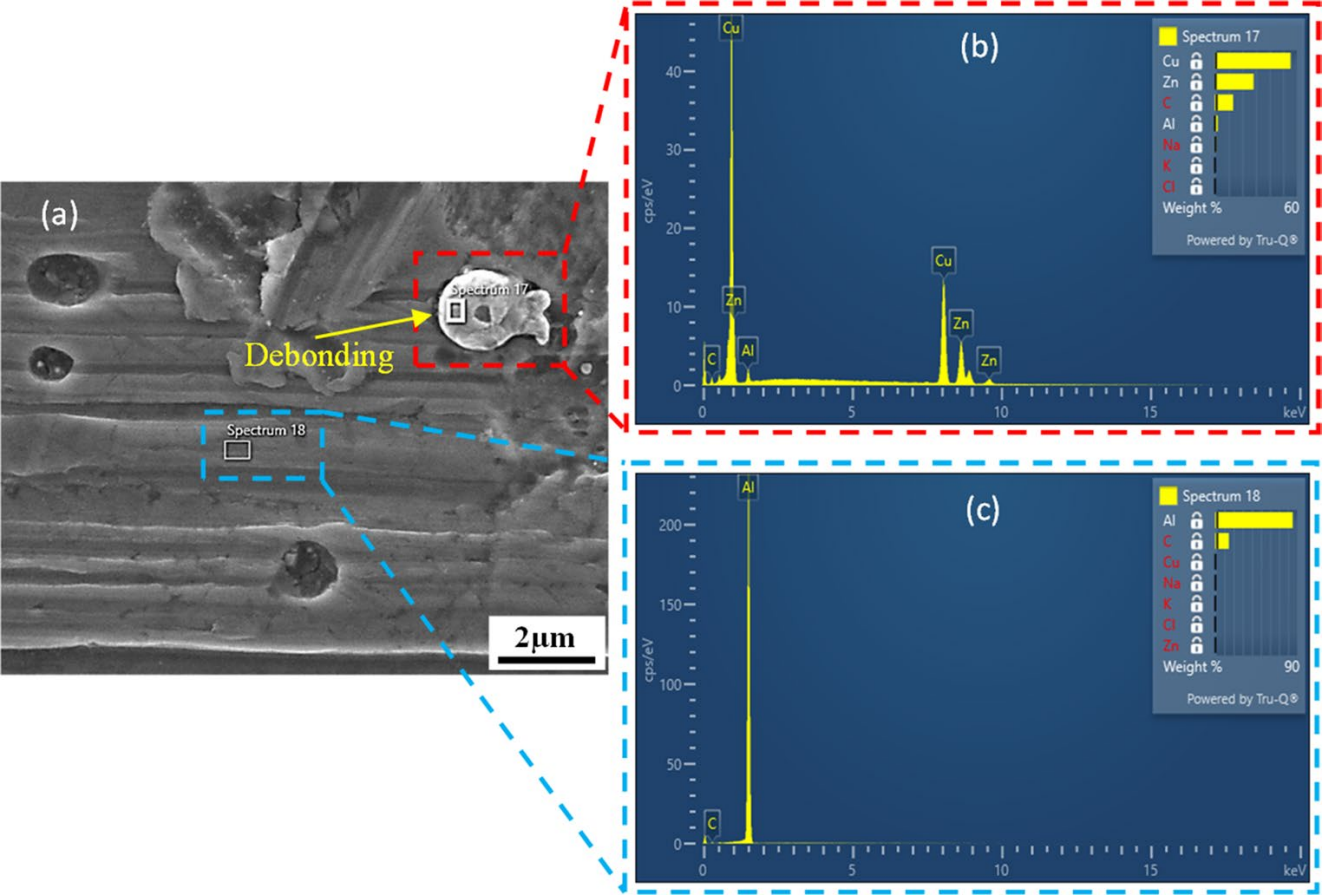
Energy-dispersive x-ray spectrometry (EDS) analysis for the sample under nucleation stage at the temperature of 85 °C. (**a**) Microscopic morphology of tensile specimen at nucleation stage. (**b**) EDS analysis results for the inclusion. (**c**) EDS analysis results for the matrix material

**Fig. 5:**
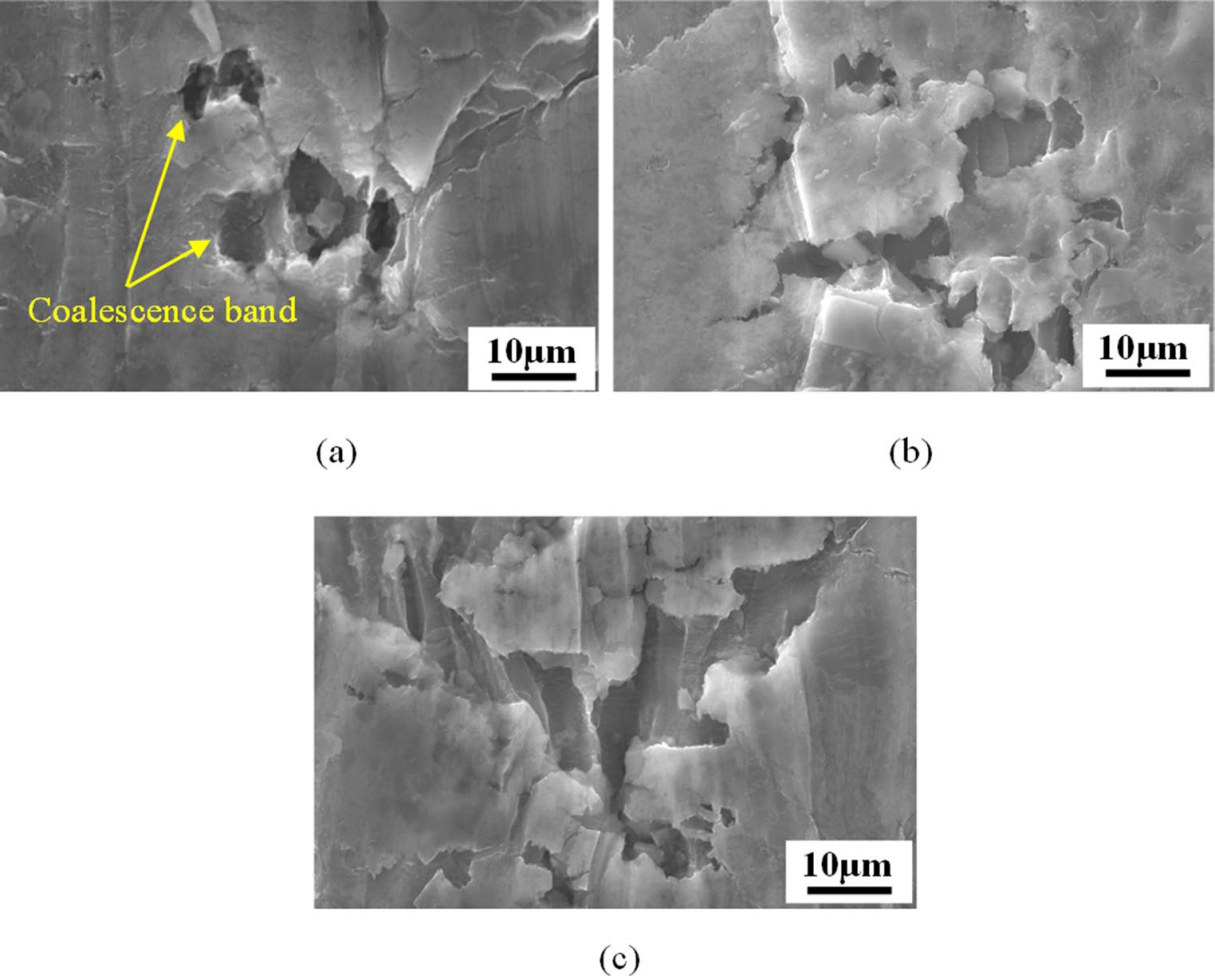
Microscopic morphology of specimen at critical stage under different temperatures. (**a**) 25 °C. (**b**) 85 °C. (**c**) 145 °C. At the critical stage, the adjacent voids coalesce together to form the coalescence band. With the increase of temperature, the size of coalescence band gradually increases

**Fig. 6:**
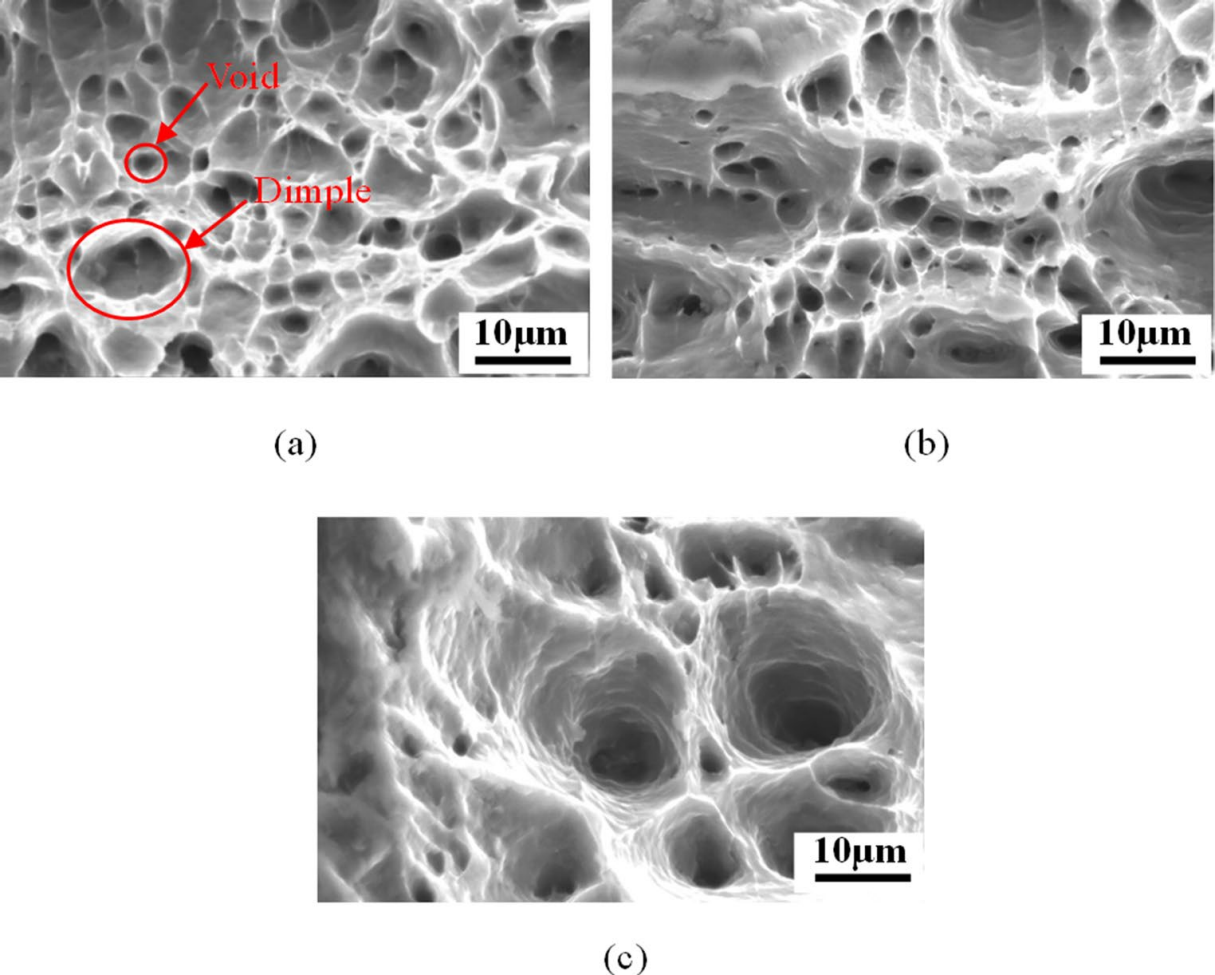
SEM images of fracture surface of tensile specimen under different temperatures. (**a**) 25 °C. (**b**) 85 °C. (**c**) 145 °C. At the fracture stage, with the increase of temperature, the size and depth of voids and dimples gradually increase

By performing EDS analysis on different areas (spectrums 17 and 18) of the sample under nucleation stage, differences in chemical composition between the inclusions and the matrix material can be distinguished. As shown in Fig. 4, spectrums 17 and 18 represent the inclusion and the matrix material, respectively. The elements of inclusion are mainly consisted of Cu and Zn, with weight% of 55.4% and 28.3%, respectively, while Al accounts for the largest weight% of 85.0% and the remaining elements are less obvious in the energy spectrum for the matrix material. With the development of plastic deformation, the original voids and nucleated voids gradually grow and expand to larger sizes. Once entering the critical stage, the inter-voids ligaments start to tear and thus the adjacent voids aggregate together to form the coalescence band and micro cracks on the material surface, as shown in Fig. 5. Thereafter, the micro cracks further extend as the deformation progresses, resulting in the occurrence of macro cracks and subsequent material fracture, as shown in Fig. 6. This phenomenon indicates that the ductile fracture is essentially a macroscopic manifestation of the evolution of micro voids.

**Fig. 7:**
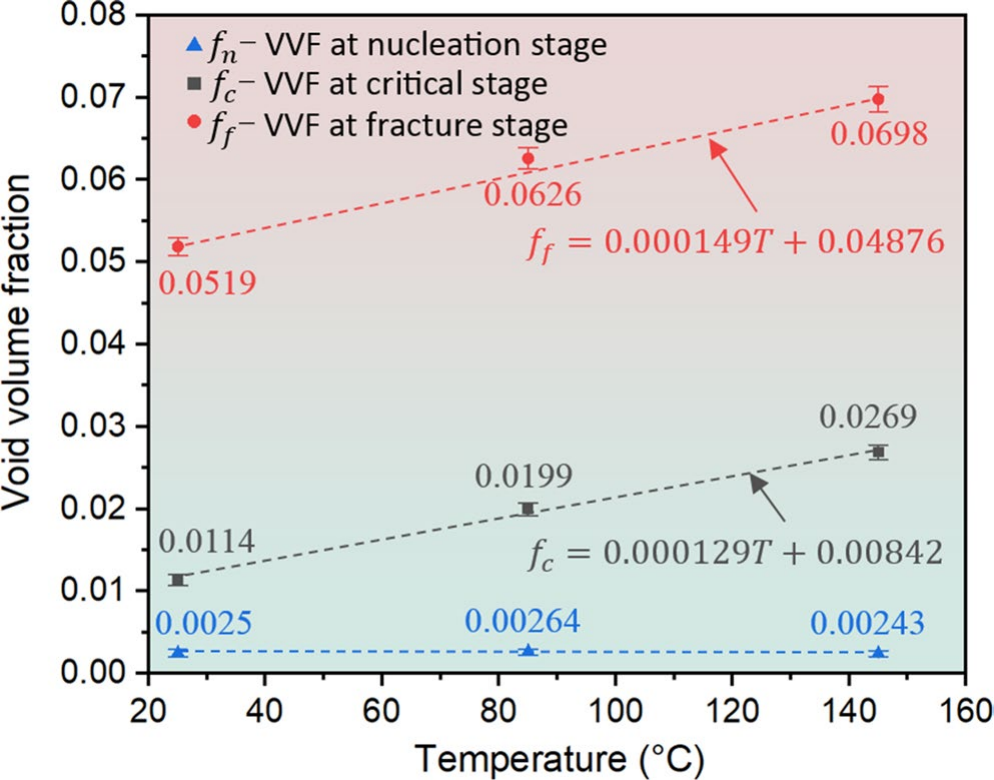
The effect of temperature on the void volume fraction (VVF) at nucleation, critical and fracture. The VVF at nucleation stage is less relevant with temperature while the variations of VVF at critical and fracture stages are positively related to the temperature

It is clear in Fig. 6 that the fracture surface of samples exhibits a cavity pattern composed of dimples and voids in correspondence with the typical characteristics of the fracture surface of ductile metallic materials [61, 62]. As is evident, the average size of dimples is larger than that of voids. This is because the dimples usually originate from initial voids or voids nucleated from second phase particles, when these voids expand to dimples (primary voids) of a certain size, due to stress concentration, new small voids (secondary voids) start to initiate around the dimples and distribute in clusters [63, 64]. The above observations reflect well the different stages of the void evolution during ductile fracture. In terms of the effect of temperature on the damage parameters ($\:{f}_{0},\:\:{f}_{n},\:\:{f}_{c},\:\:{f}_{f})$,$\:{f}_{0}$ is related to the inherent porosity of the material and is less influenced by temperatures [65]. $\:{f}_{n}$ is dependent on the degree of deformation at the yield point, since the strain shows nearly the same value at the onset of plastic deformation under different temperatures, there is no clear temperature dependency of nucleation behaviour for AA1050 samples. Regarding $\:{f}_{c}$ and $\:\:{f}_{f}$, it has been shown that the temperature exerts considerable effect on these two parameters, as shown in Fig. 7, and this temperature effect was also reported in [66].

As shown in Fig. 5, the damage degree becomes more severe and the size of coalescence band gets larger for the samples at higher elevated temperature. It can be observed from Fig. 6 that with the increase of temperature the size and depth of dimples and voids gradually increase. Similar findings were highlighted in [67, 68]. This is because higher level of ductility can be achieved at higher temperature, which prolongs the+ duration of void growth and postpones the appearance of void aggregation, thereby enlarging the average volume of voids and dimples.

### Determination and validation of Gurson–Tvergaard–Needleman (GTN) damage parameters in in-situ tensile test

As discussed above, the value of $\:{f}_{0}$ (= 0.00054) is not relevant to temperature and thus specified from the void volume fraction at 25 °C. On the other hand, the variations of $\:{f}_{c}$ and $\:{f}_{f}$ are clearly a function of temperature. The stress-strain diagram provides a direct correlation between the variation in void volume fraction and the propagation of tensile deformation at different temperatures. Thus, the void volume fraction at nucleation ($\:{f}_{n})$, critical ($\:{f}_{c}$)and fracture ($\:{f}_{f}$) stages can be identified by using ImageJ to quantitatively analyse the proportion of voids in the SEM pictures taken when the flow stress reaches the yield point, maximum tensile stress and failure point, respectively. At each temperature condition, three SEM images for each stage of void evolution are evaluated to ensure the repeatability of experimental results. The points corresponding to the determination of the void volume fraction at different stages are marked in Fig. 9. The initial void volume fraction can be determined by scanning the sheet surface prior to deformation to evaluate the proportion of initial voids and is determined as 0.00054. The void volume fraction at fracture stage can be quantified by scanning the fracture surface of sample. By analysing the sizes and distribution of voids and dimples on the fracture surface, the internal void volume fraction at the final stage of deformation can be effectively captured. The void volume fraction values and their variations at nucleation, critical and fracture stages are presented in Fig. 7. Obviously, the variations of $\:{f}_{c}$ and $\:{f}_{f}$ are positively related to the temperature ($\:T$), that is, higher temperatures promote the increase of $\:{f}_{c}$ and $\:{f}_{f}$. In addition to four damage parameters, other parameter values used in GTN model are listed in Table 2.

**Table 2:** The parameters of GTN damage model [38]

$\:{q}_{1}$	$\:{q}_{2}$	$\:{q}_{3}$	$\:{\epsilon\:}_{N}$	$\:{S}_{N}$
1.5	1	2.25	0.3	0.1

**Fig. 8:**
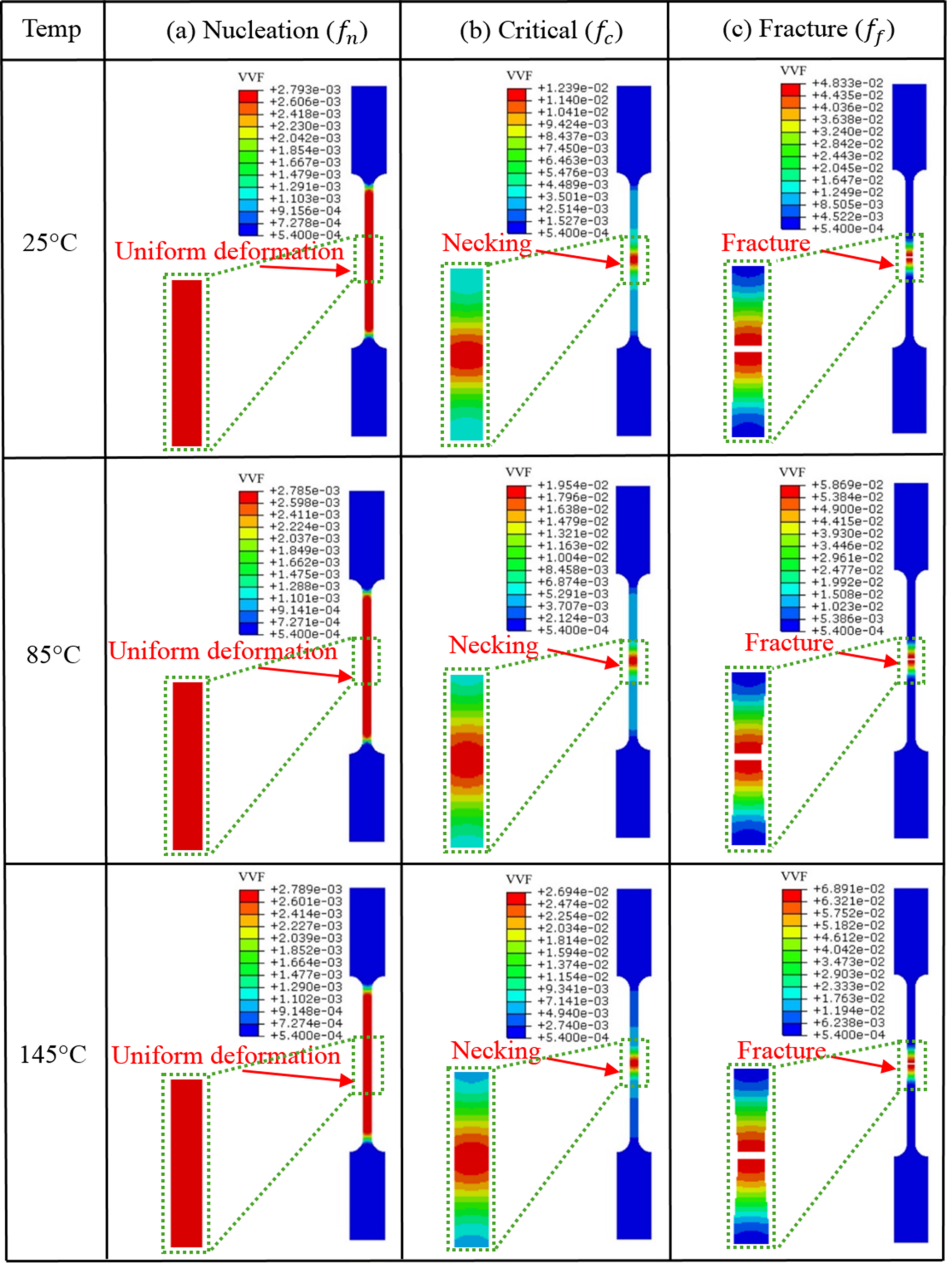
Void volume fraction (VVF) distribution of tensile simulation under different temperature conditions (25 °C, 85 °C and 145 °C) at different deformation stages. (**a**) Uniform plastic deformation stage. (**b**) The moment of necking. (**c**) The moment of fracture. This shows the high correlation between VVF evolution and damage accumulation under different temperatures

**Fig. 9:**
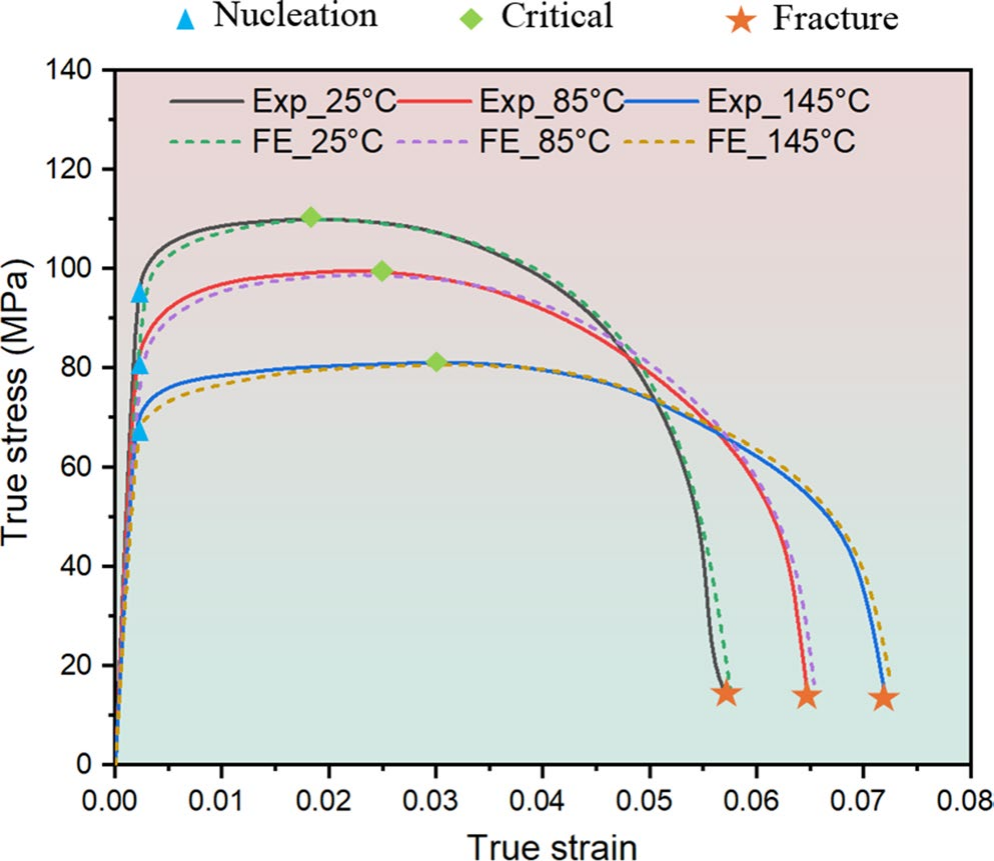
Stress-strain curves between experiment and FE under different temperatures. This shows the validity and accuracy of determined Gurson–Tvergaard–Needleman (GTN) damage parameters in predicting the fracture behaviour of material under tensile loading at different temperatures

To evaluate the reliability and validity of experimentally obtained GTN damage parameters, the tensile simulations with implementation of designated damage parameters were undertaken to study the void volume fraction (VVF) evolution and fracture behaviour of the samples at different temperatures. Figure 8 shows the VVF distribution of tensile simulation at different stages at different tensile testing temperatures of 25 °C, 85 °C and 145 °C, respectively. It is obvious that VVF exhibits similar distribution pattern during tensile loading at different temperatures. To analyse the correlation between the VVF evolution and damage accumulation, the tensile simulation at 145 °C is used as an example. As can be seen from Fig. 8, the deformation mainly takes place in the gauge region. When the VVF increases from 0.00054 to 0.0025, the sample enters uniform deformation stage and the VVF shows a uniform distribution. Microscopically, the new voids start to nucleate from inclusions or second phase particles, as shown in Fig. 3. Macroscopically, the plastic deformation is in the initial stage and the material is under a low damage condition. When the VVF reaches 0.02694, which is slightly larger than $\:{f}_{c}$, the sample undergoes inhomogeneous deformation and the VVF shows a clear localised distribution, as shown in Fig. 8 (Critical stage). At this stage, microscopically, the inter-voids ligaments rupture and thus neighbouring voids coalesce together, as shown in Fig. 5. Macroscopically, the deformation concentrates in the central area and the necking phenomenon occurs. When the VVF reaches 0.06891, the damage accumulates in the necking region and the material loses its ability to withstand mechanical loads. Microscopically, the size of voids and dimples extend to the maximum. Macroscopically, fracture appears in the middle of sample. The above observation substantiates the high correlation between VVF evolution and damage propagation and suggests that different deformation stages (yielding, necking and fracture) are activated by specific damage parameters during tensile simulation. Figure 9 presents the comparison of stress-strain curves obtained from experiment and FE at different temperatures. It is noticed that the simulation results show a good agreement with the experimental results, which indicates that the determined GTN damage parameters have a high degree of accuracy to capture the VVF evolution under loading and to predict the fracture behaviour of material under tensile loading at different temperatures.

### FE modelling of fracture in incremental sheet forming (ISF) processes

**Fig. 10:**
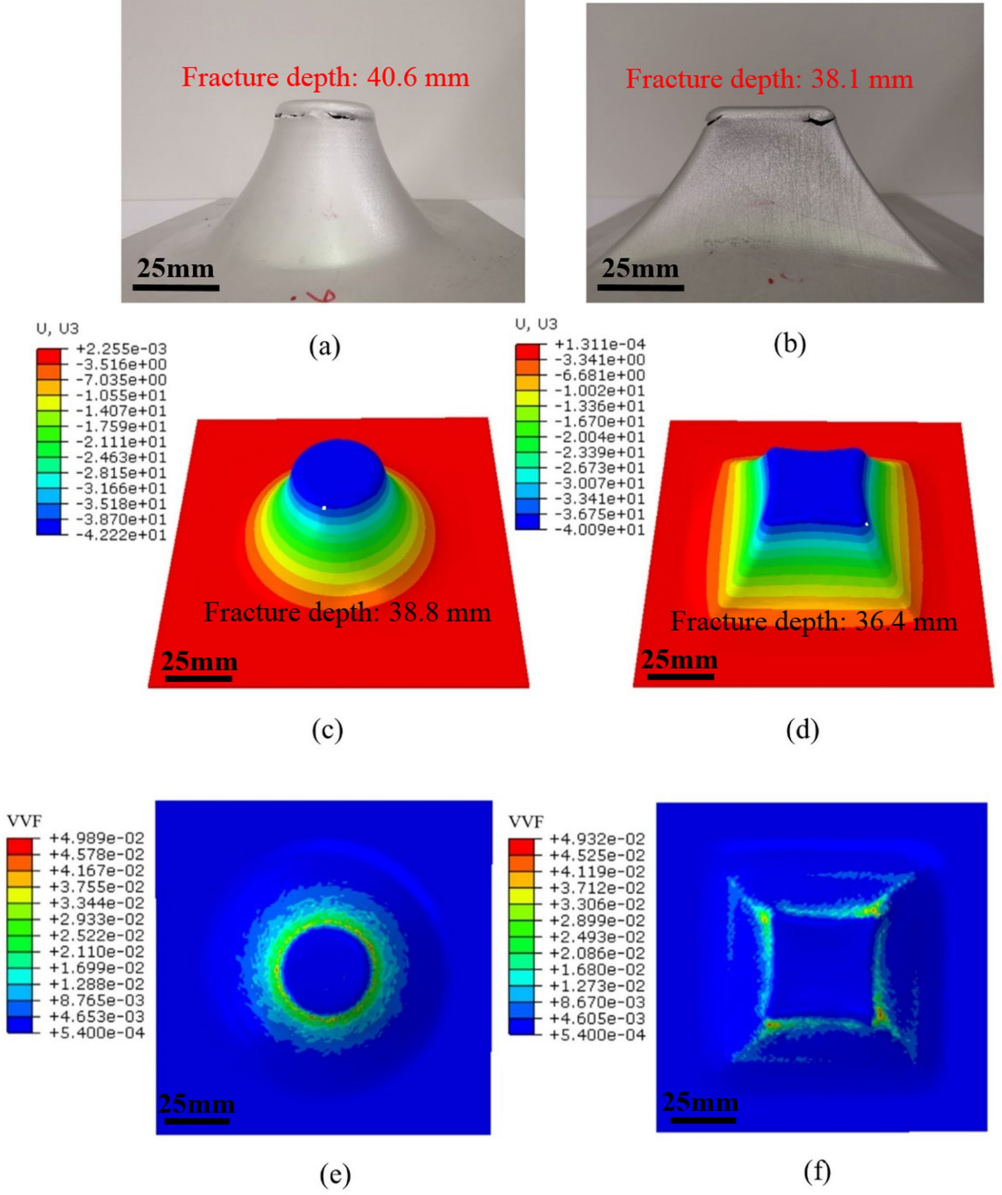
Fracture depths between experiment and FE at spindle speed of 0 rpm. (**a**) and (**b**) Formed truncated cone and truncated pyramid samples. (**c**) and (**d**) FE forming depths prediction of the truncated cone and truncated pyramid. (**e**) and (**f**) Void volume fraction (VVF) distribution at spindle speed of 0 rpm for truncated cone and truncated pyramid, respectively

Based on the microscopic analysis of void evolution, $\:{f}_{c}$ and $\:{f}_{f}$ are specified as a function of temperature to capture the void evolution and damage accumulation of the material at different temperatures. In this work, the Nahshon and Hutchinson expression shown in Eq. (4) is applied to incorporate the effect of shear on damage evolution in ISF process. This equation introduces a parameter, $\:{k}_{\omega\:}$, which governs the damage rate under shear loading. A $\:{k}_{\omega\:}$ value of 3 was adopted in this study based on findings from previous research [35]. To evaluate the accuracy of this $\:{k}_{\omega\:}$ value in predicting fracture depth relative to experimental results, finite element simulations of the hyperbolic truncated cone ISF process were performed. The simulations tested different $\:{k}_{\omega\:}$ values, including 2.5, 3, and 3.5, at a spindle speed of 0 rpm. Table 3 presents the fracture depths predicted by FE model with using different $\:{k}_{\omega\:}$ values. The results clearly demonstrate that $\:{k}_{\omega\:}$ significantly influences the prediction of fracture depth. Compared with experimental results shown in Fig. 10(a), lower $\:{k}_{\omega\:}$ values result in a marginal overestimation of fracture depth after experimental fracture occurrence, while higher $\:{k}_{\omega\:}$ values lead to an underestimation of the fracture depth prior to experimental fracture occurrence. Clearly more detailed calibration is needed to derive the correct value of $\:{k}_{\omega\:}$ for more accurate prediction.

**Table 3:** FE-predicted fracture depths with using different values of $\:{k}_{\omega\:}$

$\:{k}_{\omega\:}$	2.5	3	3.5
Fracture depth (mm)	40.9	38.8	37.2

**Fig. 11:**
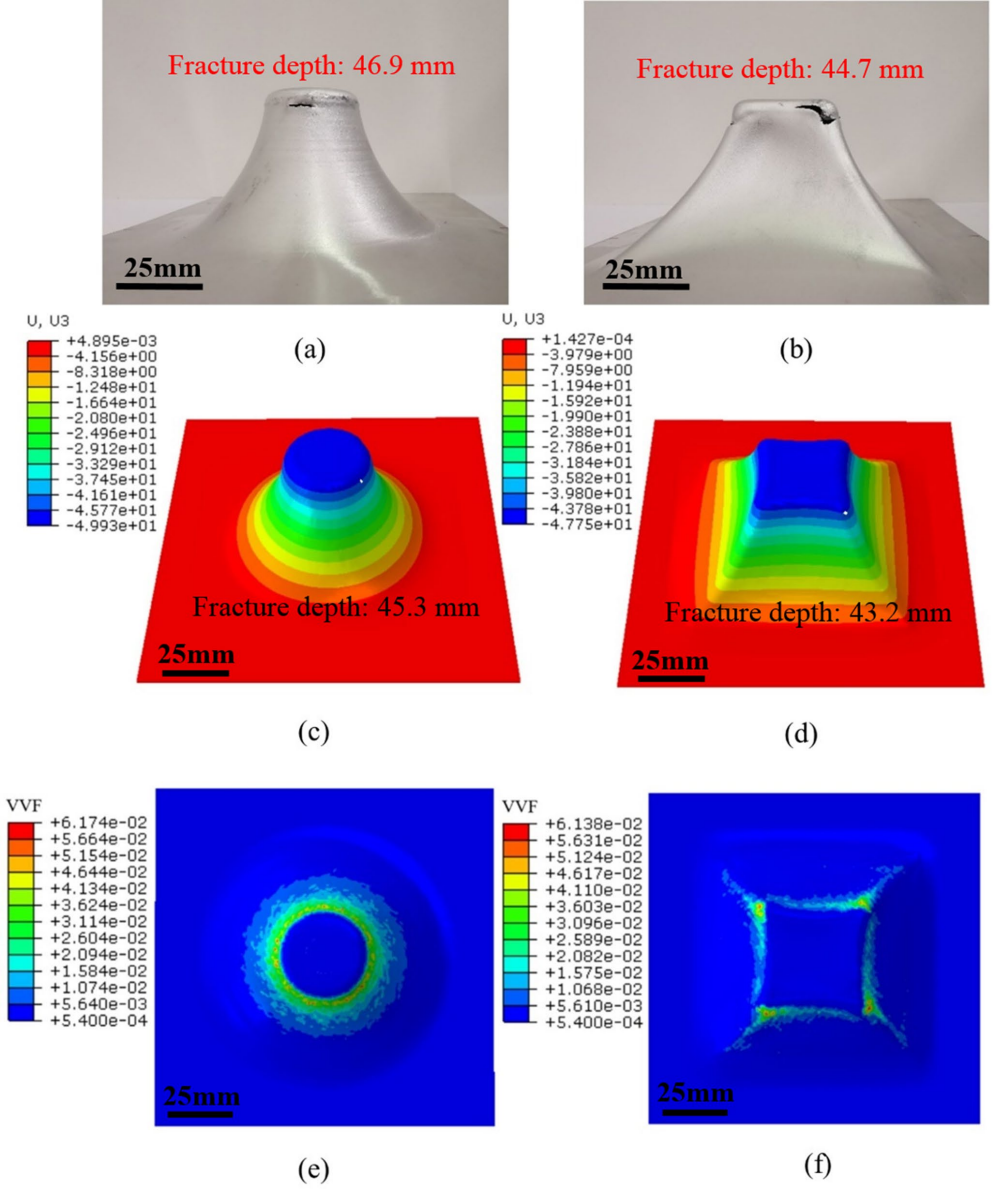
Fracture depths between experiment and FE at spindle speed of 2,500 rpm. (**a**) and (**b**) Formed truncated cone and truncated pyramid samples. (**c**) and (**d**) FE forming depths prediction of the truncated cone and truncated pyramid. (**e**) and (**f**) Void volume fraction (VVF) distribution at spindle speed of 2,500 rpm for truncated cone and truncated pyramid, respectively

**Fig. 12:**
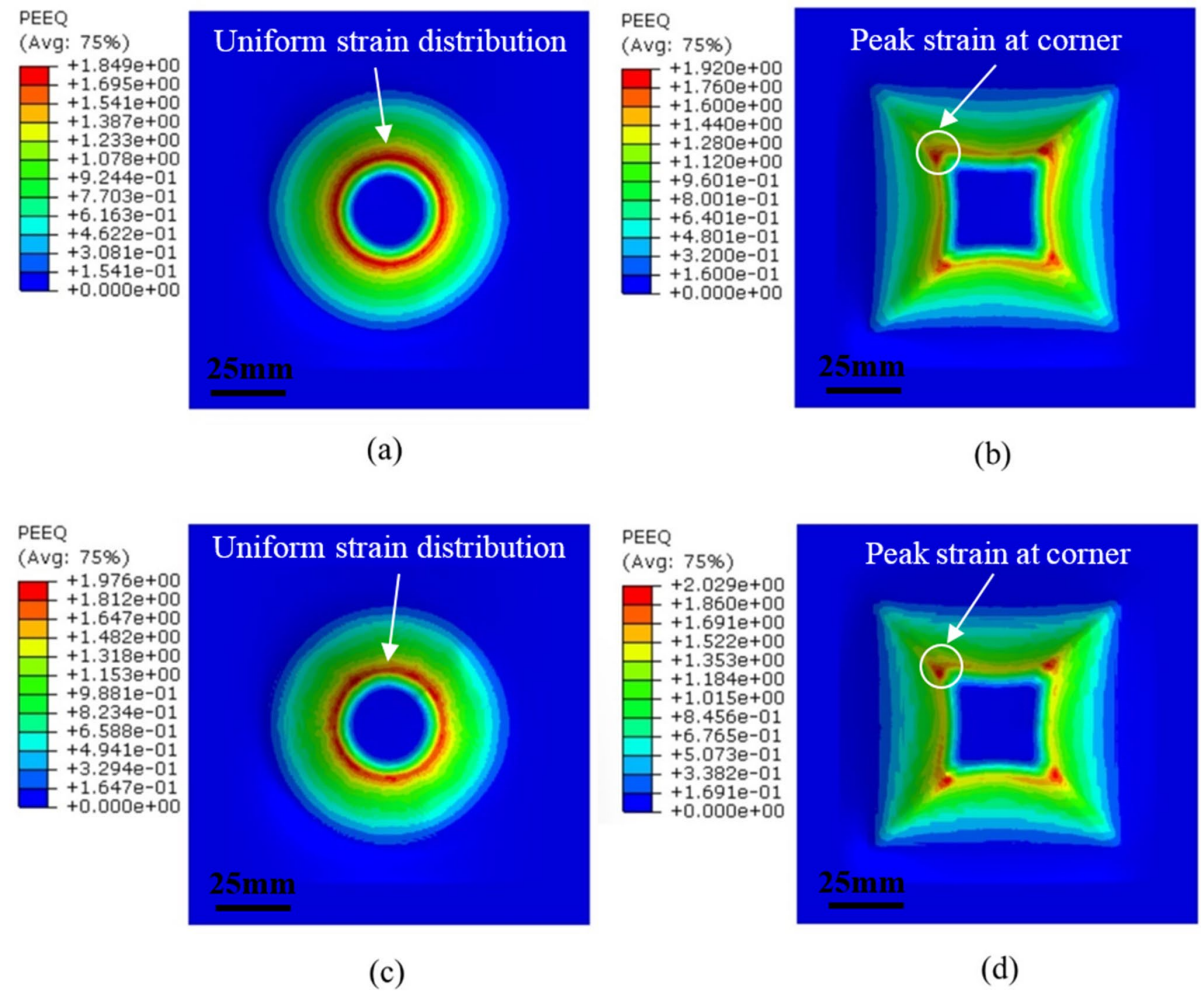
Equivalent plastic strain (PEEQ) distribution of incremental sheet forming (ISF) simulation at different spindle speeds. (**a**) and (**c**) Truncated cone part at 0 rpm and 2,500 rpm, respectively. (**b**) and (**d**) Truncated pyramid part at 0 rpm and 2,500 rpm, respectively. This shows the uniform strain distribution and peak strain at corner for truncated cone and truncated pyramid, respectively

To validate the applicability of GTN model with consideration of temperature effect in ISF, the determined GTN parameters and temperature-dependent VVF function are implemented into FE to evaluate the fracture behaviour of AA1050 under different forming conditions. Figures 10 and 11 show the comparison of the fracture depth between experiment and FE simulation at spindle speed of 0 rpm and 2,500 rpm, respectively. It can be observed that the fracture initiates at the transition area between the inclined forming wall and under-deformation base fillet for both truncated cone and truncated pyramid. This is due to the fact that the plastic deformation and degree of stretching increase with the forming depth during ISF, producing the largest value of strain at transition area which is thus characterised as the place where fracture is most likely to happen [46, 69]. However, the specific facture location occurred at a random position of the transition area for the truncated cone, whilst it always appeared at the corners for the truncated pyramid. This observation was also indicated by the VVF distribution as shown in Fig. 10(e) and (f) in the case for the truncated cone and truncated pyramid at room temperature without significant frictional heating. The same VVF distributions due to spindle speed at elevated temperatures are also shown in Fig. 11(e) and (f). As compared to the results shown in Fig. 10(e) and (f), higher VVF values appear at a random position at the location of maximum depth of the truncated cone (Fig. 11(e)) and the corner position of the pyramid (Fig. 11(f)). This is because the pyramid corners subjected to biaxial tension have higher level of plastic strain as compared to the plane strain condition of the truncated cone shape [69], as can be seen in Fig. 12(b) and (d), the peak strain at the corner position provides a clear indication of the location of fracture initiation. In terms of truncated cone, a plane strain state acts as deformation mode during the whole process [70], leading to a uniform strain distribution at transition area, as shown in Fig. 12(a) and (c).

It is also clear that although under the same processing conditions the fracture depth of the truncated pyramid is always lower than that of the truncated cone. This can be attributed to the different fracture strain between plane strain and biaxial tension states. As revealed in [23], the stress triaxiality under biaxial tension is higher than that under plane strain condition, and there is negative correlation between fracture strain and stress triaxiality, that is, higher stress triaxiality is accompanied by lower fracture strain. Thus, the damage of the truncated pyramid occurs earlier than that of the truncated cone. 

**Fig. 13:**
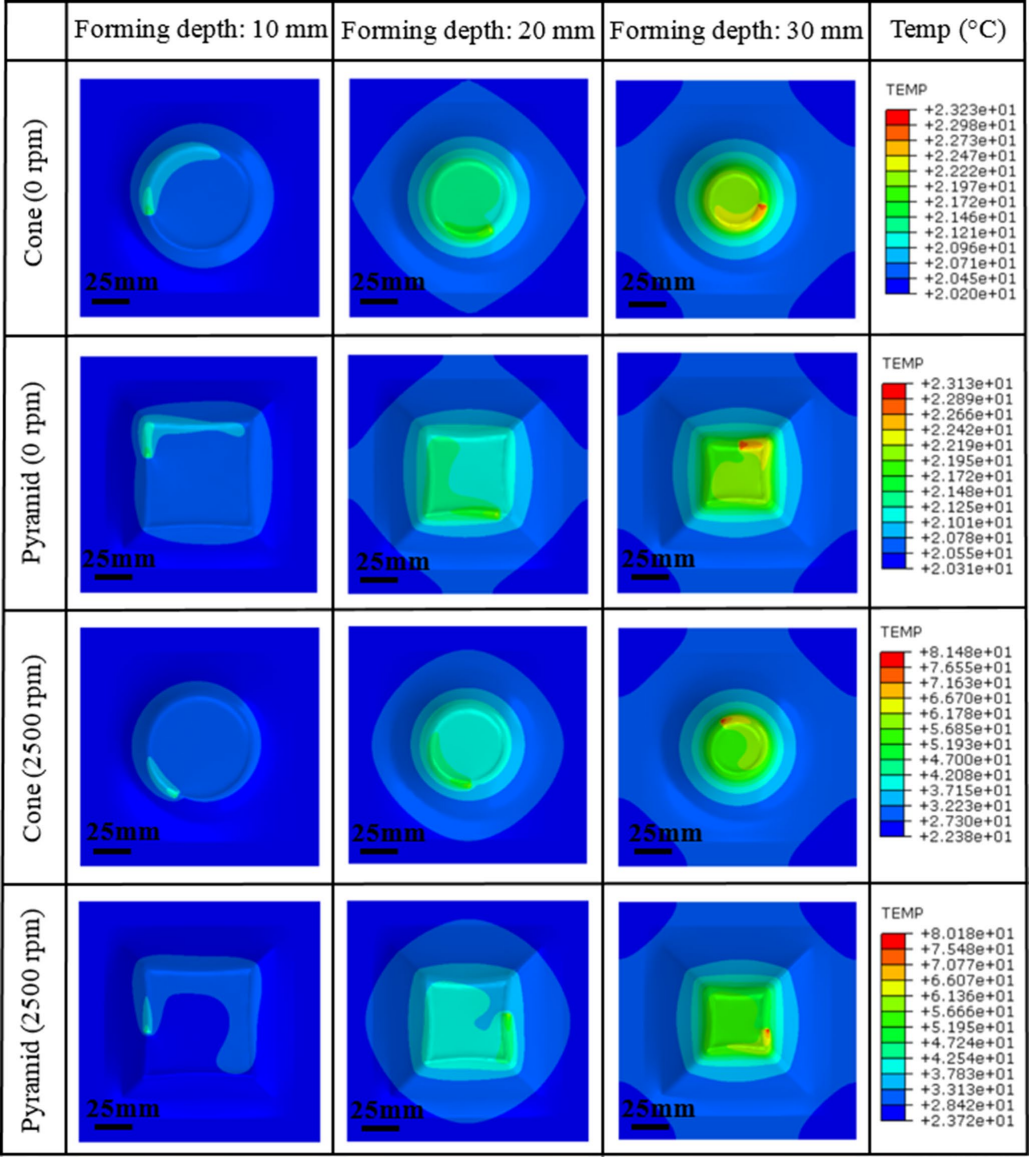
Temperature distribution of truncated cone and truncated pyramid at 0 rpm and 2,500 rpm at different depth during incremental sheet forming (ISF). This shows the spindle speed has a significant effect on temperature increase in ISF

**Fig. 14:**
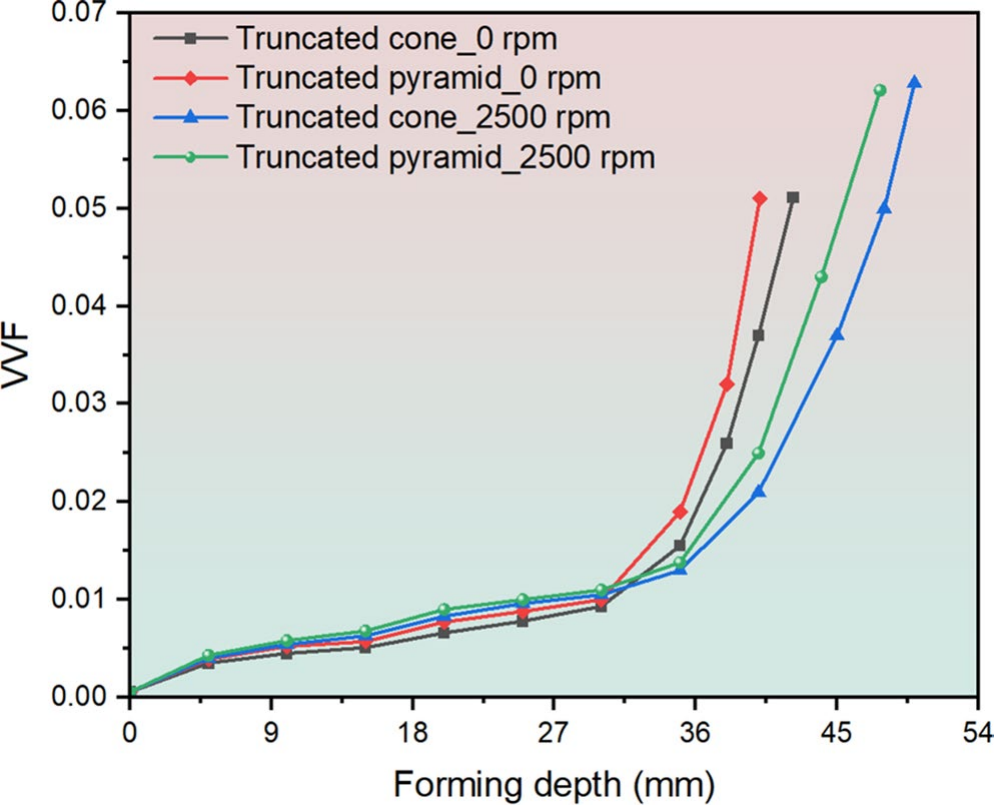
Evolution of void volume fraction (VVF) at 0 rpm and 2,500 rpm for the truncated cone and truncated pyramid during incremental sheet forming (ISF). The results reflect the effect of spindle speed on VVF evolution. As the spindle speed increases, the maximum VVF value increases, leading to delay of fracture occurrence and improved formability

As the spindle speed increases from 0 rpm to 2,500 rpm, an obvious formability improvement can be achieved, and the fracture depth increases from 40.6 mm to 46.9 mm and from 38.1 mm to 44.7 mm for the truncated cone and pyramid, respectively. This is because the higher spindle speed considerably contributes to temperature rise, as shown in Fig. 13, the forming temperature increases gradually as the process proceeds, and the temperature distribution exhibits an evident ‘comet’ shape and is highly localised around tool movement trajectory. Based on the temperature-dependent VVF functions, higher forming temperature leads to an increase in the maximum VVF value. As shown in Fig. 14, for both truncated cone and pyramid parts, the VVF reaches a larger value at spindle speed of 2,500 rpm as compared to the case at 0 rpm. Formation of fracture is delayed and the formability is improved in the case of 2,500 rpm spindle speed.

**Fig. 15:**
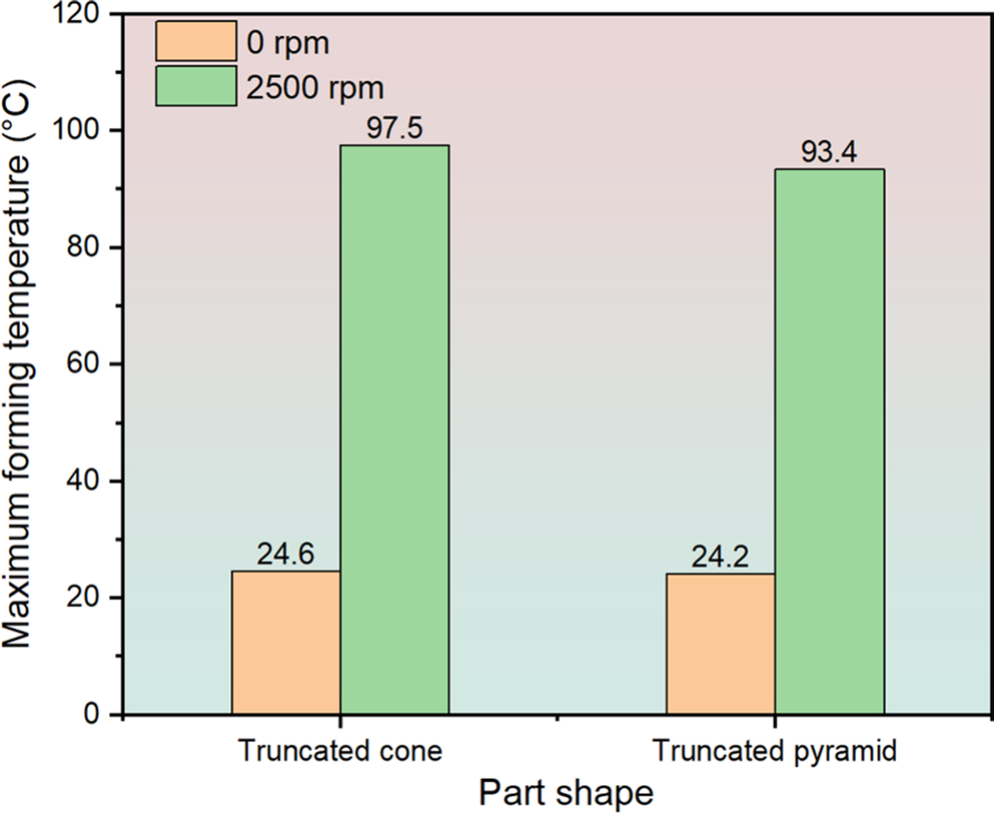
Maximum forming temperatures during incremental sheet forming (ISF) of truncated cone and truncated pyramid. This shows the spindle speed plays an important role in temperature variation

**Fig. 16:**
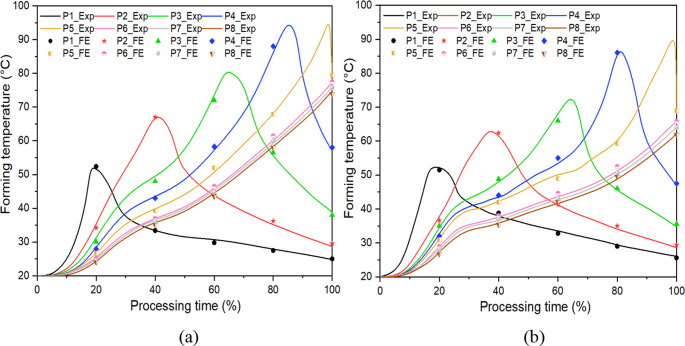
Comparison of temperature variation of tracking positions between experiment and FE simulation at rotation speed of 2,500 rpm. (**a**) Truncated cone part. (**b**) Truncated pyramid part

Figure 15 shows the maximum forming temperatures at different spindle speeds for the truncated cone and pyramid. As the spindle speed increases from 0 rpm to 2,500 rpm, the maximum forming temperature increases from 24.6 °C to 97.5 °C and from 24.2 °C to 93.4 °C for the truncated cone and pyramid, respectively, which reflects the significant role of spindle speed in temperature rise. To validate the accuracy of the FE simulated forming temperatures, a comparison between FE predictions and thermocouple measurements was conducted. As can be seen in Fig. 16, the variations of forming temperatures show a good agreement between FE simulations and experimental results. The forming temperatures of specific tracking positions gradually increase up to the peak value followed by a downward trend for P1 to P5, while it keeps increasing until the end of process for P6 to P8. This is because when the heat source is approaching the tracking point, the tracking point is subjected to increasing thermal effect; in turn, when the heat source is moving away from the tracking point, reduced thermal effect imposes on the tracking point and thus results in a temperature drop. The above observations including fracture depth, fracture position and formability enhancement of both the truncated cone and pyramid can be well reproduced in the FE results, as shown in Figs. 10 and 11, which indicates that the experimentally determined GTN damage parameters and temperature-dependent VVF functions have a high degree of accuracy and give an accurate evaluation of fracture behaviour with varying temperatures induced by spindle speeds during ISF processing.

## Conclusions

This work investigates the ductile fracture behaviour of AA1050 during incremental sheet forming (ISF) based on Gurson–Tvergaard–Needleman (GTN) model with consideration of the temperature effect. The correlation between void evolution and damage accumulation at different temperatures is revealed by conducting microscopic characterisation in in-situ tensile test. The validity of determined GTN damage parameters and temperature-dependent void volume fraction (VVF) functions are evaluated by FE simulation and experimental testing. The main conclusions can be drawn as follows:

1. From in-situ tensile test and microscopic analysis, it is confirmed that the void evolution of AA1050 follows the mechanism of nucleation, growth, coalescence and microcrack. The inclusion-matrix debonding captured at the onset of plastic deformation, the coalescence band obtained at the beginning of inhomogeneous deformation, and the distinctions in the size and distribution between dimples and voids can reflect the process of material deformation and concomitant fracture from a microscopic point of view.
2. By characterising the GTN damage parameters at different temperatures, it is found that void volume fraction at nucleation stage ($\:{f}_{n}$) is less sensitive to temperature (It is obvious in the case of the initial stage ($\:{f}_{0}$)). The void volume fractions at the critical and fracture stages ($\:{f}_{c}$ and $\:{f}_{f}$) exhibit a much higher sensitivity to the temperature variation. This is because $\:{f}_{0}$ is related to the inherent porosity of the material and $\:{f}_{n}$ is determined by the degree of deformation at the yield point which does not show clear temperature dependency. On the other hand, $\:{f}_{c}$ and $\:{f}_{f}$ correspond to the occurrence of necking and fracture which is delayed with increasing temperature. The validity of GTN damage parameters determined at different temperatures is confirmed by a good agreement between stress-strain curves obtained from FE simulations and experimental flow curves.
3. The FE simulation of micro in-situ tensile testing gives a good correlation between void evolution and damage accumulation. At the initial stage of tensile testing, the VVF shows a homogeneous distribution, reflecting the uniform deformation between yielding and necking, and when the VVF reaches $\:{f}_{c}$ and subsequently $\:{f}_{f}$, necking and fracture occur successively. This indicates that the ductile fracture is essentially a macroscopic manifestation of the evolution of micro-voids.
4. An extended GTN model is developed to incorporate the damage parameters at different temperatures and the temperature-dependent VVF functions, which is implemented to evaluate the deformation and fracture behaviour at varying forming temperatures in ISF to a metallic material for the first time. FE simulation results show that the extended model enables good predictions of fracture depth and position and gives a good reflection of the role of forming temperature in formability improvement, and captures well the effect of strain states on forming limit under different ISF processing conditions.
5. The proposed extended GTN model using the new temperature-dependent VVF functions is generic in nature to unravel the ductile fracture mechanisms of sheet material deformation under elevated temperature. It is applicable to other sheet and even bulk metal forming processes under non-isothermal deformation conditions as long as the process specific GTN parameters and the temperature-dependent VVF functions can be accurately determined. Such an approach is easy to implement and validate. It is an area expected of considerable growth.
6. The FE simulation results show that the quantification of the shear effect is an important factor in GTN modelling of fracture in ISF processing. Further work is needed for more detailed investigation and accurate calibration of the shear related parameter to achieve improved precision in predicting fracture occurrence under different ISF processing conditions. In future work, fracture locus models such as the Wierzbicki fracture criterion could be examined alongside the GTN framework to assess their suitability for practical ISF applications in terms of prediction accuracy and computational efficiency.

## Data Availability

Data can be made available with institution’s approval.
